# Movement and VIP Interneuron Activation Differentially Modulate Encoding in Mouse Auditory Cortex

**DOI:** 10.1523/ENEURO.0164-19.2019

**Published:** 2019-09-17

**Authors:** James Bigelow, Ryan J. Morrill, Jefferson Dekloe, Andrea R. Hasenstaub

**Affiliations:** 1 Coleman Memorial Laboratory, University of California, San Francisco, San Francisco, California 94143; 2 Neuroscience Graduate Program, University of California, San Francisco, San Francisco, California 94143; 3 Department of Otolaryngology–Head and Neck Surgery, University of California, San Francisco, San Francisco, California 94143

**Keywords:** information, interneurons, locomotion, optogenetics, vasoactive intestinal peptide

## Abstract

Information processing in sensory cortex is highly sensitive to nonsensory variables such as anesthetic state, arousal, and task engagement. Recent work in mouse visual cortex suggests that evoked firing rates, stimulus–response mutual information, and encoding efficiency increase when animals are engaged in movement. A disinhibitory circuit appears central to this change: inhibitory neurons expressing vasoactive intestinal peptide (VIP) are activated during movement and disinhibit pyramidal cells by suppressing other inhibitory interneurons. Paradoxically, although movement activates a similar disinhibitory circuit in auditory cortex (ACtx), most ACtx studies report reduced spiking during movement. It is unclear whether the resulting changes in spike rates result in corresponding changes in stimulus–response mutual information. We examined ACtx responses evoked by tone cloud stimuli, in awake mice of both sexes, during spontaneous movement and still conditions. VIP^+^ cells were optogenetically activated on half of trials, permitting independent analysis of the consequences of movement and VIP activation, as well as their intersection. Movement decreased stimulus-related spike rates as well as mutual information and encoding efficiency. VIP interneuron activation tended to increase stimulus-evoked spike rates but not stimulus–response mutual information, thus reducing encoding efficiency. The intersection of movement and VIP activation was largely consistent with a linear combination of these main effects: VIP activation recovered movement-induced reduction in spike rates, but not information transfer.

## Visual Abstract

**Figure:**
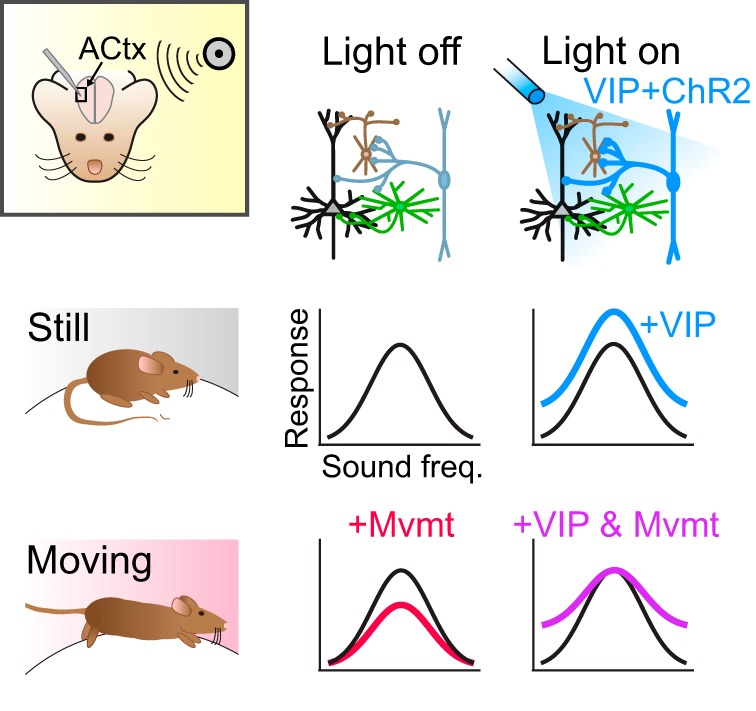

## Significance Statement

The ability of the brain to represent information about the sensory environment is heavily influenced by the behavioral state of the animal. In visual cortex, it is well known that locomotor activity enhances visual stimulus processing. By contrast, the present study found that sound processing in auditory cortex is degraded during locomotor activity. Whereas enhanced stimulus processing in visual cortex is thought to depend on VIP^+^ interneuron activation, optogenetic activation of VIP^+^ interneurons in auditory cortex failed to improve stimulus processing. These findings imply that circuitry activated during movement has opposite influences on stimulus processing in visual and auditory cortices. Such differences could reflect a resource allocation shift during movement favoring spatial perception in service of the navigational needs of the animal.

## Introduction

It is now widely accepted that information processing in sensory cortex is highly sensitive to behavioral state (Busse et al., 2017). Numerous studies have documented that the ability of visual cortex (VCtx) neurons to represent visual stimuli is enhanced during movement (Mvmt), as reflected by elevated stimulus-evoked spiking, increased stimulus–response mutual information, increased encoding efficiency, and decreased noise correlations among neuron pairs (Dadarlat and Stryker, 2017). In parallel with these changes in firing patterns, movement is associated with improved visual stimulus detection at the behavioral level (Bennett et al., 2013), as well as instantiation of several forms of lasting adult cortical plasticity (Kaneko et al., 2017). A specific disinhibitory circuit activated by subcortical motor signals appears to be responsible for this movement-related enhancement of encoding (Lee et al., 2013), as follows: ascending projections from the midbrain locomotor region activate basal forebrain cholinergic neurons, which in turn activate inhibitory interneurons in VCtx expressing vasoactive intestinal peptide (VIP^+^). VIP^+^ neurons are key constituents of a disinhibitory circuit in which they preferentially suppress other inhibitory interneurons, especially those expressing somatostatin (Sst^+^), thus elevating pyramidal cell firing rates (Pi et al., 2013). Importantly, optogenetic activation and genetic blockade experiments have found that VIP^+^ interneuron activity is both necessary and sufficient to account for the effects of movement in VCtx studied thus far, including augmented visually evoked responses (Fu et al., 2014) and instantiation of adult cortical plasticity (Fu et al., 2015).

Disinhibition of pyramidal neurons by VIP–Sst circuit activation has been observed elsewhere in the brain, including the auditory cortex (ACtx; Pi et al., 2013). As in VCtx, VIP^+^ interneurons in ACtx are activated during movement (Fu et al., 2014). Paradoxically, however, studies of ACtx generally find that most neurons exhibit diminished stimulus-evoked firing rates during movement (Schneider et al., 2014, Zhou et al., 2014). These outcomes imply that, in contrast to VCtx, VIP^+^ activation does not fully reproduce the effects of movement in ACtx. It is possible that VIP^+^-mediated disinhibition of evoked responses in ACtx resulting from movement may be overwhelmed by other sources of inhibition, such as elevated inhibitory interneuron activity initiated by projections from motor cortex (Nelson et al., 2013, Schneider et al., 2014). If so, strengthening the influence of VIP^+^ interneurons through optogenetic activation could recover or reverse the loss in stimulus-evoked spike rates associated with movement.

Nevertheless, it is not presently understood whether the differential changes in firing rate resulting from movement and VIP^+^ activation produce corresponding changes in information transfer. As numerous studies have documented, cell- or population-level differences in overall firing rates between conditions may not coincide with differences in information representation, let alone in the same direction. For instance, optogenetic activation of interneurons expressing parvalbumin and somatostatin in ACtx reduced spike rates without changing stimulus–response mutual information, suggesting increased encoding efficiency (Phillips and Hasenstaub, 2016). It follows that stimulus information in ACtx could be preserved despite reduced stimulus-evoked spike rates during movement, reflecting increased encoding efficiency. Consistent with this possibility, ACtx neurons in cats performing a spatial auditory task exhibited significant increases in spatial sensitivity despite a reduction in overall firing rates (Lee and Middlebrooks, 2011; see also Christensen and Pillow, 2017). Alternatively, recent behavioral experiments observed reduced sound detection accuracy during movement (McGinley et al., 2015a), suggesting that acoustic information in ACtx may not be fully preserved during movement for exploitation by feedforward networks supporting behavioral choice. It similarly remains to be determined whether the increased evoked firing rates resulting from VIP^+^ interneuron activation have any impact on information representation. Hypothetically, if VIP^+^ interneuron activation increases the information carried about stimuli, this could potentially offset the decrease in information transfer resulting from movement.

We examined these questions by quantifying ACtx encoding of tone cloud stimuli in awake, head-fixed mice during spontaneous movement and stationary conditions. VIP^+^ interneurons were optogenetically activated on half of the trials, permitting independent analysis of the consequences of locomotor activity and VIP^+^ interneuron activity, as well as their intersection. We focus our analysis on measures capturing stimulus-driven spike rates, the information (bits) carried by these spikes, and encoding efficiency (bits per spike).

## Materials and Methods

### Subjects and surgical preparation

All procedures were approved by the Institutional Animal Care and Use Committee at the University of California, San Francisco. A VIP-Cre knock-in line was used to target opsins to VIP^+^ cells (stock #010908, The Jackson Laboratory), which drives expression of Cre in VIP^+^ interneurons of the cortex, hippocampus, olfactory bulb, and suprachiasmatic nuclei, as well as several discrete midbrain and brainstem regions (Taniguchi et al., 2011). This Cre line was then crossed with the Ai32 line (C57BL/6 background; stock #012569, The Jackson Laboratory), which encodes the light-gated depolarizing cation channel channelrhodopsin-2 (ChR2) conjugated to enhanced yellow fluorescent protein (eYFP), after a floxed stop cassette under the CAG promoter. A total of nine adult animals (four male, five female) served as subjects (age range, 13–16 weeks). Animals were housed in groups of two to five under a reverse light/dark cycle (8:00 A.M. lights off, 8:00 P.M. lights on). All experiments were performed during the dark phase. All surgical procedures were conducted under isoflurane anesthesia with perioperative analgesics (lidocaine, meloxicam, and buprenorphine) and monitoring. A custom stainless steel headbar was affixed to the cranium above the right temporal lobe with dental cement, after which subjects were allowed to recover for at least 2 d. On the day of the experiment, a brief craniotomy procedure (∼20–30 min) produced a small opening (∼1–2 mm diameter) centered above ACtx (∼2.5–3.5 mm posterior to bregma and under the squamosal ridge) within a window opening of the headbar. The craniotomy was promptly sealed with silicone elastomer (Kwik-Cast, World Precision Instruments). The animal was observed until ambulatory (∼5–10 min) and allowed to recover for a minimum of 2 h before recording commenced (Dadarlat and Stryker, 2017).

### Treadmill and movement detection

**Figure 1.**
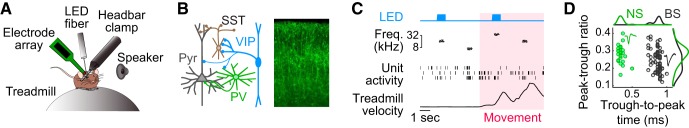
Extracellular recording and optogenetic manipulation of ACtx neurons in freely moving mice. ***A***, Mice are head fixed atop a spherical treadmill to permit movement but constrain head position for extracellular physiology. A window in the headbar provides access to ACtx of the right hemisphere for extracellular recording with linear multielectrode arrays and optogenetic manipulation via LED fiber optic cables. Sounds are presented to the contralateral ear through an electrostatic speaker. ***B***, Optogenetic activation of VIP^+^ inhibitory interneurons in ACtx. Left, Schematic of prominent connections among cortical inhibitory interneurons and pyramidal neurons. Sst^+^ and Pvalb^+^ interneurons predominantly connect to pyramidal dendrites and somata, respectively. VIP^+^ interneurons most prominently connect to Sst^+^ interneurons. Thus, VIP^+^ interneuron activation by blue light (right) thus tends to disinhibit pyramidal neurons by reducing Sst^+^ interneuron-mediated inhibition. Right, Control of VIP^+^ interneurons via blue light was accomplished by crossing VIP-Cre mice with Ai32 mice to express channelrhodopsin-2 in Cre-expressing VIP interneurons. Histology figure from ACtx in a representative Ai32/VIP-Cre mouse expressing eYFP-tagged ChR2 (green) in VIP^+^ interneurons. Scale bar, 100 μm. ***C***, Stimuli comprised 500 ms tone clouds spanning 4–64 kHz presented in pseudorandom order. Optogenetic stimulation was delivered for half of all trials in pseudorandom order. Simultaneous recording from 2–15 neurons was permitted by 32-channel linear multielectrode arrays. Treadmill velocity was stored for off-line analysis, yielding both still and movement periods. ***D***, Each unit was characterized by its peak/trough ratio (peak height divided by trough depth) and trough-to-peak time. A sharply bimodal distribution of trough-to-peak times permitted straightforward identification of NS (putative inhibitory) and BS (putative excitatory) neurons. Inset plots show example NS and BS waveforms (mean ± SD).

The animal was affixed by a headbar clamp atop a free-spinning spherical treadmill as depicted in Figure 1*A* (Dombeck et al., 2007; Niell and Stryker, 2010; Dadarlat and Stryker, 2017; Phillips et al., 2017a; Hoglen et al., 2018; Morrill and Hasenstaub, 2018). A polystyrene ball (diameter, 200 mm) was placed inside a polystyrene bowl (diameter, 250 mm) with a single air inlet at the bottom. An optical USB mouse, placed ∼1–3 mm from the surface of the ball, was used to transmit movement signals to the data acquisition system using custom driver software. Each animal was habituated to the treadmill for at least 1–2 h before the day of the experiment.

### Electrophysiology

Recordings were conducted inside a sound attenuation chamber (Industrial Acoustics Company). The silicone plug filling the craniotomy was removed, and a single-shank, multichannel linear probe (32 channels, 25 or 50 μm site spacing; NeuroNexus) was slowly lowered into cortex using a motorized microdrive (FHC). Before lowering the probe, but after initial positioning, the craniotomy was filled with 2% agarose to stabilize the brain surface. After reaching a depth of 710–850 μm below the first observation of action potentials, the probe was allowed to settle for at least 20 min before commencement of the experiment. Extracellular voltage signals were stored for off-line analysis with an RHD2000 evaluation system (Intan Technologies) at a sample rate of 30 kHz. Other experimental events such as optogenetic and acoustic stimulation epochs were stored concurrently by the same system. The raw voltage traces were filtered off-line (0.3–5 kHz), after which a custom spike-sorting program (MATLAB) was used to assign spike waveforms to individual units based on clustering in three-dimensional waveform feature space (e.g., projections onto principal components, spike times). Auto-correlation and cross-correlation analysis, refractory period analysis, and cluster isolation statistics were used to support single-unit assignments. Each penetration yielded 2–15 single units. A total of 96 units were obtained from 12 penetrations in nine animals (2 penetrations were made in three animals). Similar to previous publications (Seybold et al., 2015; Rummell et al., 2016; Phillips et al., 2017b), units were classified as narrow spiking (NS; putative inhibitory) or broad spiking (BS; putative excitatory) on the basis of a clear bimodal distribution of waveform peak-to-trough durations (Fig. 1*D*; NS, <600 μs; BS, ≥600 μs). Unless otherwise specified, each of the comparisons between conditions below reflect group analyses of the NS (*n* = 25) and BS (*n* = 71) unit subpopulations combined across subjects.

Optogenetic stimulation was delivered by a 470 nm LED (Mightex) coupled to an optical fiber (400 μm diameter, 0.39 numerical aperture; Thorlabs) positioned on the surface of the dura by a micromanipulator immediately adjacent to the electrode array. Optogenetic stimulation spanned 200 ms before and 100 ms after the tone clouds (800 ms total), with a 50 ms linear ramp to reach full power at onset. LED power was adjusted for each experiment (range, 0.2–1.6 mW) to achieve an average firing rate change of ∼50% across channels.

### Auditory stimuli

Auditory stimuli comprised 500 ms tone clouds centered at 20 frequencies spanning 4–64 kHz (Fig. 1*C*). The tone clouds were constructed by randomly generating a 50 ms tone pip within 0.2 octave of the specified frequency at 25 ms intervals. Thus, two pips were present at any instant during the stimulus other than the first and last 25 ms, which contained only a single pip. Stimuli were generated in MATLAB (MathWorks) and delivered using Psychophysics Toolbox Version 3 (Kleiner et al., 2007) through a free-field electrostatic speaker (ES1, Tucker-Davis Technologies) driven by an external sound card (Quad Capture, Roland) at a sample rate of 192 kHz and a mean intensity of 60 ± 5 dB. The speaker was positioned ∼15–20 cm from the left (contralateral) ear. Sound levels were calibrated using a Brüel & Kjær model 2209 meter and a model 4939 microphone. Each frequency was repeated 50 times with and without optogenetic stimulation, with an intertrial interval of 2 s (total experiment time, ∼90 min).

### Data analysis

Movement trials were defined by mean treadmill displacement during the 500 ms tone cloud period in excess of a threshold defined for each experiment as determined by the noise floor of the optical mouse tracker (∼1 cm/s). Because of the spontaneous nature of the movement condition, the four possible combinations of movement and VIP interneuron activation (Still, Mvmt, Still + VIP, Mvmt + VIP) occurred on different numbers of trials. To offset the potential impact of different trial counts in the analysis below, a subsampling procedure was used to equate the number of repetitions for each stimulus (tone cloud frequency) in each condition. This was done by identifying the stimulus/condition intersection with the fewest trials and selecting an equivalent number of trials from the remaining stimuli/conditions at random. To minimize the dependence of the results on any particular subsampling choice, the procedure was repeated 100 times, and the mean across iterations was included in the analyses reported below. Due to limited sampling, the original 20 tone cloud frequencies were collapsed into groups of four adjacent frequencies, yielding five frequency bins.

Mutual information was calculated according to the following equation:

$$I\left(X;Y\right)=\underset{x\in X}{\sum }\underset{y\in Y}{\sum }p\left(x,y\right)*log_{2}\left(\frac{p\left(x,y\right)}{p\left(x\right)*p\left(y\right)}\right).$$

X and Y represent the stimulus (tone cloud frequency) and response distributions (sum of evoked spike counts during the stimulus period), respectively. The terms p(x) and p(y) specify the probabilities of observing a given stimulus and response, respectively, and p(x,y) specifies their joint probability (i.e., the probability of the response, given the stimulus). Intuitively, mutual information values (expressed in bits) reflect the degree to which responses are consistent within frequency and distinctive across frequencies. A shuffling procedure was used to correct for potential bias introduced by limited sampling of the stimulus and response probability distributions (Panzeri et al., 2007). Stimulus–response pairs were randomly shuffled (1000 iterations) to produce a distribution of null information values. The mean of the null distribution was then subtracted from the observed mutual information estimate to eliminate that expected by chance. Encoding efficiency (bits/spike) was quantified by dividing the bias-corrected mutual information value by the mean spike count across trials. All mutual information analyses reported below were unadjusted for spontaneous firing rates.

Firing rate correlations were calculated for each simultaneously recorded neuron pair (*n* = 441) using single-trial spike rates during the stimulus window. Because few pairs of simultaneously recorded NS neurons were available in our sample, this analysis was agnostic to putative neuron class. Total correlations, which are assumed to reflect the sum of signal and noise correlations, were defined by the Pearson correlation coefficient between spike count vectors of neuron pairs across all trials in their preserved order. Signal correlations, which are thought to reflect shared variability in stimulus preferences, were obtained by shuffling trial order while preserving the stimulus labels. Noise correlations, which reflect coordinated activity resulting from factors extrinsic to the stimuli, are then obtained by subtracting signal from total correlation estimates.

### Statistical analysis

Unless otherwise indicated, significant differences between conditions were assessed using two-tailed Wilcoxon signed-rank (WSR) tests. In the majority of analyses, each condition was examined for differences in stimulus-driven spike rates, stimulus information, and encoding efficiency. In such cases, the Benjamini–Hochberg procedure was implemented to control false discovery rate (FDR) individually for each unit type (*q* = 0.05, adjustment for the three tests; Benjamini and Hochberg, 1995). A similar procedure was implemented for analyses of coordinated network variability, except that the FDR adjustment reflected only two tests (signal and noise correlations). Each test is accompanied by an effect size estimate defined by $r=\frac{z}{\sqrt{2n}}$, where z is the WSR test statistic (using the approximate method) and n is the number of paired comparisons. Mean and median values are provided for each condition with 95% confidence intervals (CIs) estimated with 1000 bootstrap iterations. For comparison with previous work in VCtx (Dadarlat and Stryker, 2017), we also report the mean percentage difference (plus 95% CI) between conditions for analyses involving firing rate and information values, and the mean difference (plus 95% CI) between correlation coefficients for analyses of coordinated network variability.

## Results

### Summary of movement trials

Across recordings, the mean fraction of trials in which mice were engaged in movement was 0.19 (CI, 0.15–0.24), which is comparable to previous studies of spontaneous movement in mice (Dombeck et al., 2007; Keller et al., 2012). Mean movement velocity was 7.83 cm/s (CI, 6.81–8.92) for trials classified as Mvmt and 0.18 (CI, 0.14–0.23) for trials classified as Still.

### Movement reduces stimulus-evoked spike rates, stimulus information, and encoding efficiency

**Figure 2.**
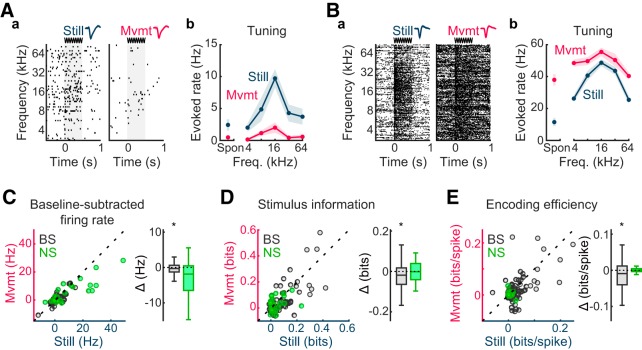
Movement reduces stimulus-related spike rates, information content, and encoding efficiency in ACtx. ***A***, Example unit exhibiting suppressed firing during movement. ***a***, Raster plots depicting responses to tone clouds in the absence of (Still) and during movement (Mvmt). Each tick represents an action potential; each raster line reflects activity on a single trial. Unit waveform samples (∼1.8 ms) from each condition (mean ± SD) appear above the rasters. The stimulus period is represented by gray shading in the raster plots and by the sine waveform above the plots. ***b***, Frequency–tuning curve plots for each condition (mean ± SEM), including spontaneous (Spon) firing rates. ***B***, Example unit exhibiting elevated firing during movement. Subplot organization and labeling as in ***A***. ***C–E***, Scatterplot summaries of stimulus-evoked firing rates, stimulus information, and encoding efficiency during Still and Mvmt conditions (*n* = 71 BS units, *n* = 25 NS units). A difference box plot (Δ) appears to the right of each scatterplot representing Mvmt values subtracted from Still values. Box plot mid lines indicate medians, outer lines indicate 25th and 75th percentiles, and whiskers indicate the range of data points excluding outliers. The consequences of movement were heterogeneous for our unit samples. On balance, movement was associated with reduced spike rates in excess of spontaneous rates for both BS and NS units, but the difference only reached statistical significance for BS units. Stimulus information (bits) as well as encoding efficiency (bits/spike) were significantly reduced in BS units, but remained unchanged for NS units. *p < 0.05, signed-rank tests.

Spontaneous firing rates were defined for all conditions as the mean spike count (per trial) within a window equal to the stimulus period before LED onset (i.e., 700–200 ms before tone cloud onset). The influence of movement on spontaneous and evoked firing rates was heterogeneous in our unit sample. Example units showing diminished and elevated firing rates during movement are shown respectively in Figure 2, *A* and *B*. The raster subplots in Figure 2, *Aa* and *Ba*, depict peristimulus spike times for each trial and condition, and the frequency–tuning curves in Figure 2, *Ab* and *Bb*, summarize mean spontaneous and evoked firing rates for each tone cloud frequency. Across our unit sample, spontaneous firing rates were unexpectedly elevated during movement trials [*p* < 10^−5^, *r* = 0.344, WSR test; Still: mean = 6.51 (CI, 5.14–8.12), median = 3.63 (CI, 3.21–5.19); Mvmt: mean = 8.97 (CI, 6.93–11.18), median = 4.32 (CI, 3.56–6.31); mean change from Still = +38% (CI, +24 to +53%)], a finding that held when NS [*p* < 10^−4^, *r* = 0.561, WSR test; Still: mean = 13.35 (CI, 9.49–17.71), median = 11.88 (CI, 6.54–17.05); Mvmt: mean = 19.97 (CI, 13.77–25.60), median = 15.57 (CI, 10.41–27.64); mean change from Still = +50% (CI, +29 to +72%)] and BS [*p* = 0.006, *r* = 0.229, WSR test; Still: mean = 4.11 (CI, 3.28–5.06), median = 3.22 (CI, 2.46–3.67); Mvmt: mean = 5.09 (CI, 4.01–6.21), median = 3.65 (CI, 2.71–4.35); mean change from Still = +24% (CI, +9% to +39%)] subpopulations were considered separately. We were unable to obtain clear evidence for the dependence of these outcomes on cortical depth, although variation across recordings in the dorsal–ventral and caudal–rostral angles at which probes entered ACtx precluded rigorous assignment of units to cortical layer. A subtle increase in firing rate also persisted into the stimulus period for NS units such that a borderline effect was observed for raw mean evoked rates [*p* = 0.054, *r* = 0.272, WSR test; Still: mean = 23.13 (CI, 16.79–30.18), median = 19.65 (CI, 12.06–33.62); Mvmt: mean = 25.62 (CI, 18.52–32.79), median = 23.94 (CI, 13.86–35.16); mean change from Still = +11% (CI, −4% to +25%)]. The difference was nonsignificant for BS units [*p* = 0.176, *r* = 0.113, WSR test; Still: mean = 5.17 (CI, 4.15–6.20), median = 4.02 (CI, 2.81–5.17); Mvmt: mean = 5.48 (CI, 4.39–6.60), median = 4.10 (CI, 2.65–5.73); mean change from Still = +6% (CI, −4% to +16%)].

Previous studies have generally reported suppressed spontaneous spiking activity in ACtx during movement, although several exceptions and inconsistencies are worth noting. In an initial study, movement was associated with consistently suppressed firing of BS (putative excitatory) units and elevated firing of NS (putative inhibitory) units across superficial and deep layers (Schneider et al., 2014). A subsequent study observed predominant suppression in neurons (unlabeled) that was limited to superficial layers, with no effect of movement in layer 4 (Zhou et al., 2014). By contrast, a study using two-photon imaging to track the activity of superficial layer labeled VIP^+^ interneurons found no effect of movement on the spontaneous activity of unlabeled neurons (Fu et al., 2014). Yet another study observed significantly elevated layer 5 multiunit firing during movement (McGinley et al., 2015a). Studies examining spontaneous firing rates during movement in VCtx have also yielded discrepancies (Shimaoka et al., 2018), with some studies reporting no significant change during movement (Niell and Stryker, 2010; Polack et al., 2013; Fu et al., 2014), others reporting significant increases (Ayaz et al., 2013; Erisken et al., 2014), and still others reporting mixed effects (Saleem et al., 2013). Considered together, these outcomes suggest that spontaneous firing during movement in sensory cortex may be more diverse than initially recognized. At present, the distribution of these changes among neuron subpopulations and across cortical layers remains incompletely understood.

We defined the stimulus-evoked response as the mean firing rate during the tone cloud period minus the mean firing rate during the spontaneous period (Vinck et al., 2015; Nieto-Diego and Malmierca, 2016). Mutual information values were computed using the raw spike rates (unadjusted for spontaneous activity). Under these definitions, a significant decrease in stimulus-evoked spike rates (Fig. 2*C*) was observed for BS units [*p* = 0.023, *r* = 0.191, WSR test; Still: mean = 1.06 (CI, 0.20–1.93), median = 0.06 (CI, −0.16 to +0.90); Mvmt: mean = 0.39 (CI, −0.31 to +1.16), median = −0.08 (CI, −0.34 to +0.20); mean change from Still = −64% (CI, −111% to −20%)]. A similar trend was observed for NS units, but was nonsignificant after FDR correction [*p* = 0.104, *r* = 0.299, WSR test; Still: mean = 9.78 (CI, 5.01–15.51), median = 3.94 (CI, 1.13–12.52); Mvmt: mean = 5.65 (CI, 2.84–8.64), median = 2.65 (CI, −0.30 to +8.47); mean change from Still = −42% (CI, −73% to −11%)]. The diminished spike rates observed during movement coincided with a decrease in mutual information values (Fig. 2*D*) in the BS subpopulation (*p* = 0.023, *r* = 0.200, WSR test; Still: mean = 0.08 (CI, 0.06–0.11), median = 0.03 (CI, 0.01–0.08); Mvmt: mean = 0.06 (CI, 0.04–0.09), median = 0.02 (CI, 0.01–0.04); mean change from Still = −24% (CI, −47% to +2%)], with no significant difference observed in NS units [*p* = 0.907, *r* = 0.021, WSR test; Still: mean = 0.04 (CI, 0.02–0.07), median = 0.01 (CI, 0.01–0.04); Mvmt: mean = 0.03 (CI, 0.02–0.05), median = 0.03 (CI, 0.00–0.05); mean change from Still = −14% (CI, −80% to +47%)]. A corresponding loss of encoding efficiency (Fig. 2*E*) was observed for BS units [*p* = 0.023, *r* = 0.211, WSR test; Still: mean = 0.04 (CI, 0.03–0.05), median = 0.02 (CI, 0.01–0.03); Mvmt: mean = 0.03 (CI, 0.01–0.04), median = 0.01 (CI, 0.00–0.02); mean change from Still = −30% (CI, −57% to +1%)] but not for NS units [*p* = 0.907, *r* = 0.017, WSR test; Still: mean = 0.00 (CI, 0.00–0.01), median = 0.00 (CI, 0.00–0.01); Mvmt: mean = 0.00 (CI, 0.00–0.01), median = 0.00 (CI, 0.00–0.01); mean change from Still = −13% (CI, −142% to +107%)].

In summary, despite heterogeneity among individual neurons, on balance, movement was associated with significantly decreased stimulus-evoked spike rates, mutual information, and encoding efficiency in BS neurons. Qualitatively, these outcomes stand opposite to those obtained in VCtx, where movement is associated with increased stimulus-evoked spike rates as well as stimulus information and encoding efficiency. Quantitatively, however, the influence of movement appears to be somewhat stronger in VCtx than in ACtx, perhaps reflecting differential involvement in spatial navigation (see Discussion). According to Dadarlat and Stryker (2017), movement was associated with a 62% increase in evoked spike rates (average across all visual stimuli) and a 47% increase in bits/spike in VCtx, although no distinction was made between NS and BS subtypes in these analyses. We therefore reanalyzed the current unit sample in ACtx collapsing across neuron subtypes and obtained decreases of 47% and 29% during Mvmt trials for stimulus-evoked spike rates and bits/spike respectively. In an earlier study, Niell and Stryker (2010) reported that movement increased the number of spikes driven by the preferred visual stimulus by >200% for BS units and that a similar trend was obtained for NS units. We therefore conducted parallel analyses in our ACtx unit sample, with stimulus-evoked spike rates defined as above except that the response to the preferred tone cloud stimulus on Still trials was substituted for the average response across tone clouds. Decreases of 49% and 52% were obtained for NS and BS units, respectively.

### VIP^+^ interneuron activation increases stimulus-evoked spike rates but not stimulus information

Previous studies have documented that, as in other cortical areas, the activation of VIP^+^ interneurons in ACtx results in elevated spontaneous and evoked firing rates (Pi et al., 2013). However, whether these additional spikes contribute to stimulus information and/or encoding efficiency remains unknown. We examined these questions in VIP-Cre mice crossed with Ai32 mice to express ChR2 in VIP^+^ interneurons (Fig. 1*B*).

**Figure 3.**
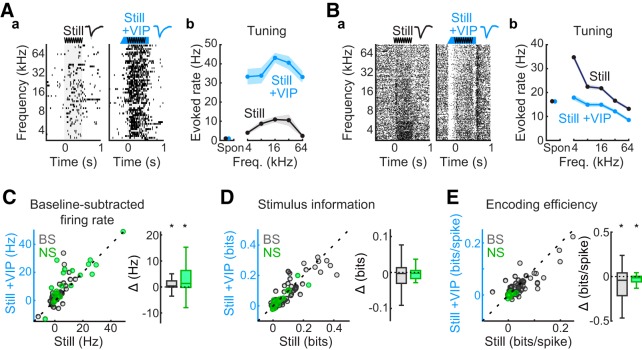
VIP^+^ interneuron activation increases spike rates but not stimulus–response mutual information in ACtx. ***A***, Example unit exhibiting elevated firing in response to VIP^+^ interneuron activation. ***a***, Raster plots depicting responses to tone clouds without (Still) and with (Still + VIP) optogenetic activation of VIP interneurons. Only trials in which subjects were not moving were included to isolate the consequences of VIP activation from those of movement. Each tick represents an action potential; each raster line reflects activity on a single trial. Unit waveform samples (∼1.8 ms) from each condition (mean ± SD) appear above the rasters. The stimulus period is represented by gray shading in the raster plots and by the sine waveform above the plots. The optogenetic activation period, including onset ramp, is indicated by the blue bar above the rasters. ***b***, Frequency–tuning curve plots for each condition (mean ± SEM), including spontaneous (Spon) firing rates. ***B***, Example unit exhibiting suppressed firing in response to VIP interneuron activation. Subplot organization and labeling as in ***A***. ***C–E***, Scatterplot summaries of stimulus-evoked firing rates, stimulus information, and encoding efficiency during Still and Still + VIP conditions (*n* = 71 BS units, *n* = 25 NS units). A difference box plot (Δ) appears to the right of each scatterplot representing Still + VIP values subtracted from Still values. Box plot mid lines indicate medians, outer lines indicate 25th and 75th percentiles, and whiskers indicate the range of data points excluding outliers. VIP^+^ interneuron activation elevated stimulus-evoked firing rates for the majority of both neuron classes but did not significantly alter information (bits) about the stimulus. Consequently, encoding efficiency (bits/spike) decreased for both unit types. *p < 0.05, signed-rank tests.

We first examined stimulus-evoked responses, information, and encoding efficiency during still trials to isolate the influence of VIP^+^ activation from the influence of movement on ACtx encoding dynamics. As expected, VIP^+^ activation resulted in elevated firing stimulus-evoked spike rates for most units (Fig. 3*A*), despite several examples with opposite responses (Fig. 3*B*). On average, a significant increase in stimulus-evoked spike rates (Fig. 3*C*) was observed for both the NS unit [*p* = 0.037, *r* = 0.318, WSR test; Still: mean = 9.78 (CI, 4.93–15.67), median = 3.94 (CI, 1.13–12.52); Still + VIP: mean = 13.93 (CI, 7.95–19.73), median = 10.36 (CI, 2.88–21.26); mean change from Still = +42% (CI, +11% to +78%)] and the BS unit [*p* < 10^−3^, *r* = 0.346, WSR test; Still: mean = 1.06 (CI, 0.23–1.95), median = 0.06 (CI, −0.16 to +0.90); Still + VIP: mean = 2.64 (CI, 1.24–4.21), median = 1.05 (CI, 0.46–1.67); mean change from Still = +149% (CI, +59% to +247%)] subpopulations. Importantly, however, the additional spikes had no significant impact on mutual information estimates (Fig. 3*D*) for either unit subpopulation [NS: *p* = 0.143, *r* = 0.207, WSR test; Still: mean = 0.04 (CI, 0.02–0.06), median = 0.01 (CI, 0.01–0.04); Still + VIP: mean = 0.03 (CI, 0.01–0.06), median = 0.01 (CI, 0.00–0.04); mean change from Still = −16% (CI, −42% to +6%); BS: *p* = 0.304, *r* = 0.086, WSR test; Still: mean = 0.08 (CI, 0.06–0.10), median = 0.03 (CI, 0.02–0.07); Still + VIP: mean = 0.08 (CI, 0.06–0.10), median = 0.03 (CI, 0.02–0.06); mean change from Still = −7% (CI, −22% to +8%)]. Consistent with the finding that information remained the same despite an increase in spike count, a significant decrease in encoding efficiency (Fig. 3*E*) was observed in each case [NS: *p* = 0.037, *r* = 0.348, WSR test; Still: mean = 0.00 (CI, 0.00–0.01), median = 0.00 (CI, 0.00–0.01); Still + VIP: mean = 0.00 (CI, 0.00–0.00), median = 0.00 (CI, 0.00–0.00); mean change from Still = −72% (CI, −125% to −29%); BS: *p* = 0.010, *r* = 0.229, WSR test; Still: mean = 0.04 (CI, 0.03–0.05), median = 0.02 (CI, 0.01–0.04); Still + VIP: mean = 0.03 (CI, 0.02–0.04), median = 0.02 (CI, 0.01–0.02); mean change from Still = −20% (CI, −38% to −3%)].

### Competing influences of movement and VIP^+^ interneuron activation offset each other

**Figure 4.**
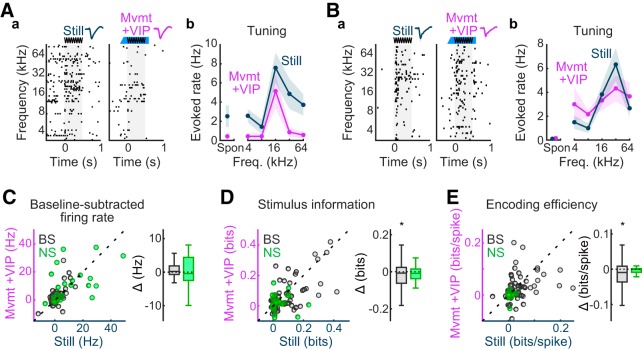
VIP^+^ interneuron activation recovers evoked spike rates, but not stimulus–response mutual information lost during movement. ***A***, Example unit for which baseline-subtracted spike rates and frequency–response functions are similar between Still and Mvmt + VIP conditions. ***a***, Raster plots depicting responses to tone clouds in the absence of (Still) and with movement and VIP^+^ interneuron activation (Mvmt + VIP). Each tick represents an action potential; each raster line reflects activity on a single trial. Unit waveform samples (∼1.8 ms) from each condition (mean ± SD) appear above the rasters. The stimulus period is represented by gray shading in the raster plots and by the sine waveform above the plots. The optogenetic activation period, including onset ramp, is indicated by the blue bar above the rasters. ***b***, Frequency–tuning curve plots for each condition (mean ± SEM), including spontaneous (Spon) firing rates. ***B***, Example unit for which baseline-subtracted spike rates are similar between Still and Mvmt + VIP conditions, despite a relatively flattened frequency–response function in the Mvmt + VIP condition. Subplot organization and labeling as in ***A***. ***C–E***, Scatterplot summaries of stimulus-evoked firing rates, stimulus information, and encoding efficiency during Still and Mvmt + VIP conditions (*n* = 71 BS units, *n* = 25 NS units). A difference box plot (Δ) appears to the right of each scatterplot representing Mvmt + VIP values subtracted from Still values. Box plot mid lines indicate medians, outer lines indicate 25th and 75th percentiles, and whiskers indicate the range of data points excluding outliers. ***C***, On average, movement and VIP^+^ interneuron activation had opposite influences on stimulus-related spike rates (Figs. 2, 3), which cancelled out when these respective influences occurred simultaneously. ***D***, ***E***, Whereas stimulus information and encoding efficiency decreased during movement for BS units, no changes were observed during VIP^+^ activation, and thus information remained low when both occurred at the same time. No significant changes were observed for NS units. *p < 0.05, signed-rank tests.

We next examined the impact of VIP interneuron activation on stimulus-evoked responses, information, and encoding efficiency within the context of trials in which the animal was moving, to uncover any potential nonlinear interaction between movement and VIP^+^ activation. Without such an interaction, we would expect to recover the reduction in spike rates associated with movement, but not stimulus–response mutual information. Indeed, such linear outcomes were largely born out in our dataset. Across the unit population, the tendency of movement to diminish spike rates summed linearly with the tendency of VIP^+^ activation to elevate spike rates, effectively cancelling each other out. Thus, no significant difference in stimulus-evoked spike rates was observed between Still and Mvmt + VIP conditions (Fig. 4*C*) for either unit subpopulation [NS: *p* = 0.946, *r* = 0.010, WSR test; Still: mean = 9.78 (CI, 4.67–15.80), median = 3.94 (CI, 1.13–12.52); Mvmt + VIP: mean = 10.12 (CI, 5.89–14.77), median = 9.08 (CI, 0.02–13.76); mean change from Still = +4% (CI, −43% to +52%); BS: *p* = 0.144, *r* = 0.123, WSR test; Still: mean = 1.06 (CI, 0.21–1.95), median = 0.06 (CI, −0.16 to +0.90); Mvmt + VIP: mean = 1.83 (CI, 0.64–3.10), median = 0.49 (CI, −0.02 to +1.38); mean change from Still = +73% (CI, −17% to +161%)]. A similar linear summation was evident in measures of information carried by these spikes (Fig. 4*D*,*E*). Thus, consistent with the observations that stimulus information and encoding efficiency were reduced in BS units by movement but were unchanged by VIP^+^ activation, significant reductions were similarly observed in bits [*p* = 0.021, *r* = 0.184, WSR test; Still: mean = 0.08 (CI, 0.06–0.11), median = 0.03 (CI, 0.02–0.07); Mvmt + VIP: mean = 0.06 (CI, 0.03–0.08), median = 0.02 (CI, 0.01–0.04); mean change from Still = −31% (CI, −57% to −6%)] and bits/spike [*p* = 0.005, *r* = 0.202, WSR test; Still: mean = 0.04 (CI, 0.03–0.05), median = 0.02 (CI, 0.01–0.03); Mvmt + VIP: mean = 0.03 (CI, 0.01–0.04), median = 0.01 (CI, 0.01–0.03); mean change from Still = −33% (CI, −68% to +1%)] during Mvmt + VIP trials. Consistent with the finding that these reductions were isolated to BS units in the Mvmt condition, the reductions were similarly nonsignificant for the NS unit subpopulation in the Mvmt + VIP condition [bits: *p* = 0.510, *r* = 0.135, WSR test; Still: mean = 0.04 (CI, 0.02–0.06), median = 0.01 (CI, 0.01–0.04); Mvmt + VIP: mean = 0.02 (CI, 0.00–0.05), median = 0.02 (CI, 0.01–0.04); mean change from Still = −43% (CI, −127% to +24%); bits/spike: *p* = 0.510, *r* = 0.166, WSR test; Still: mean = 0.00 (CI, 0.00–0.01), median = 0.00 (CI, 0.00–0.01); Mvmt + VIP: mean = 0.00 (CI, −0.01 to 0.00), median = 0.00 (CI, 0.00–0.00); mean change from Still = −96% (CI, −230% to +12%)].

Similar conclusions were generally supported by comparisons between Mvmt + VIP and Mvmt, and between Mvmt + VIP and Still + VIP conditions. Thus, stimulus-driven spike rates were significantly higher for Mvmt + VIP compared with Mvmt [NS: *p* = 0.014, *r* = 0.401, WSR test; Mvmt: mean = 5.65 (CI, 2.91–8.92), median = 2.65 (CI, −0.30 to 9.53); Mvmt + VIP: mean = 10.12 (CI, 5.85–15.08), median = 9.08 (CI, 0.02–13.76); mean change from Mvmt = +79% (CI, +34% to +136%); BS: *p* < 10^−3^, *r* = 0.317, WSR test; Mvmt: mean = 0.39 (CI, −0.30 to 1.12), median = −0.08 (CI, −0.34 to 0.22); Mvmt + VIP: mean = 1.83 (CI, 0.67–3.25), median = 0.49 (CI, −0.02 to +1.38); mean change from Mvmt = +374% (CI, +144% to +642%)]. They were similarly lower for Mvmt + VIP compared with Still + VIP, although the differences did not reach significance after FDR adjustment [NS: *p* = 0.052, *r* = 0.337, WSR test; Still + VIP: mean = 13.93 (CI, 8.43–20.28), median = 10.36 (CI, 2.88–21.26); Mvmt + VIP: mean = 10.12 (CI, 5.45–14.57), median = 9.08 (CI, 0.02–13.76); mean change from Still + VIP = −27% (CI, −51% to −4%); BS: *p* = 0.107, *r* = 0.173, WSR test; Still + VIP: mean = 2.64 (CI, 1.21–4.44), median = 1.05 (CI, 0.46–1.67); Mvmt + VIP: mean = 1.83 (CI, 0.60–3.19), median = 0.49 (CI, −0.02 to +1.38); mean change from Still + VIP = −30% (CI, −57% to −9%)]. Stimulus information remained unchanged between Mvmt + VIP and Mvmt [NS: *p* = 0.530, *r* = 0.097, WSR test; Mvmt: mean = 0.03 (CI, 0.01–0.05), median = 0.03 (CI, 0.00–0.05); Mvmt + VIP: mean = 0.02 (CI, 0.00–0.04), median = 0.02 (CI, 0.01–0.03); mean change from Mvmt = −34% (CI, −110% to +46%); BS: *p* = 0.452, *r* = 0.086, WSR test; Mvmt: mean = 0.06 (CI, 0.04–0.09), median = 0.02 (CI, 0.01–0.04); Mvmt + VIP: mean = 0.06 (CI, 0.03–0.08), median = 0.02 (CI, 0.01–0.04); mean change from Mvmt = −9% (CI, −39% to +21%)]. A significant reduction in stimulus information was expected for BS units between Mvmt + VIP and Still + VIP, whereas only borderline effects in this direction were obtained (*p* = 0.071, *r* = 0.152, WSR test; Still + VIP: mean = 0.08 (CI, 0.05–0.10), median = 0.03 (CI, 0.02–0.05); Mvmt + VIP: mean = 0.06 (CI, 0.03–0.08), median = 0.02 (CI, 0.01–0.04); mean change from Still + VIP = −26% (CI, −47% to −3%)]. The difference was nonsignificant for NS units, as expected [*p* = 0.904, *r* = 0.017, WSR test; Still + VIP: mean = 0.03 (CI, 0.01–0.06), median = 0.01 (CI, 0.00–0.04); Mvmt + VIP: mean = 0.02 (CI, 0.00–0.04), median = 0.02 (CI, 0.01–0.04); mean change from Still + VIP = −33% (CI, −132% to +45%)]. In an apparent deviation from the general trend toward linear sum effects, losses in encoding efficiency individually incurred by movement and VIP^+^ activation did not additively compound when these occurred together, as suggested by nonsignificant comparisons between Mvmt + VIP and Mvmt [NS: *p* = 0.527, *r* = 0.089, WSR test; Mvmt: mean = 0.00 (CI, 0.00–0.01), median = 0.00 (CI, 0.00–0.01); Mvmt + VIP: mean = 0.00 (CI, −0.01 to 0.00), median = 0.00 (CI, 0.00–0.00); mean change from Mvmt = −95% (CI, −289% to +68%); BS: *p* = 0.452, *r* = 0.063, WSR test; Mvmt: mean = 0.03 (CI, 0.01–0.04), median = 0.01 (CI, 0.00–0.02); Mvmt + VIP: mean = 0.03 (CI, 0.01–0.04), median = 0.01 (CI, 0.01–0.03); mean change from Mvmt = −4% (CI, −60% to +50%)], and between Mvmt + VIP and Still + VIP [NS: *p* = 0.600, *r* = 0.074, WSR test; Still + VIP: mean = 0.00 (CI, −0.00 to 0.00), median = 0.00 (CI, 0.00–0.00); Mvmt + VIP: mean = 0.00 (CI, 0.00–0.00), median = 0.00 (CI, 0.00–0.00); mean change from Still + VIP = −84% (CI, −423% to +216%); BS: *p* = 0.238, *r* = 0.099, WSR test; Still + VIP: mean = 0.03 (CI, 0.02–0.04), median = 0.02 (CI, 0.01–0.02); Mvmt + VIP: mean = 0.03 (CI, 0.01–0.04), median = 0.01 (CI, 0.01–0.03); mean change from Still + VIP = −16% (CI, −60% to +24%)].

In summary, VIP^+^ interneuron activation reliably produced an increase in stimulus-evoked spike rates without a concomitant increase in information carried about the stimulus, thereby reducing encoding efficiency. We detected a predominantly linear interaction between the effects of movement and VIP^+^ activation, such that it was possible to recover the reduction in evoked spike rates associated with movement, but not the amount or efficiency of information carried about stimuli. These outcomes extend previous findings within ACtx in suggesting that, unlike VCtx, the effects of movement in ACtx cannot be reproduced by VIP^+^ interneuron activation.

### Coordinated network variability decreases during movement

The preceding results indicate that during movement, stimulus-evoked spike rates, information carried about the stimulus, and encoding efficiency are diminished in ACtx, whereas previous studies have found that they are enhanced in VCtx. Several studies have now documented changes in VCtx network variability that likely contribute to movement-related improvements in stimulus information and encoding efficiency. Subtle decreases in signal correlations, but more substantial decreases in noise correlations have been observed between neuron pairs during movement (Erisken et al., 2014; Vinck et al., 2015; Dadarlat and Stryker, 2017). Whereas signal correlations are thought to reflect shared variability in stimulus preferences, noise correlations are thought to stem from factors extrinsic to the visual stimuli. Thus, a differential reduction in noise correlations relative to signal correlations may increase the efficiency with which these stimuli are encoded by VCtx neuronal populations.

**Figure 5.**
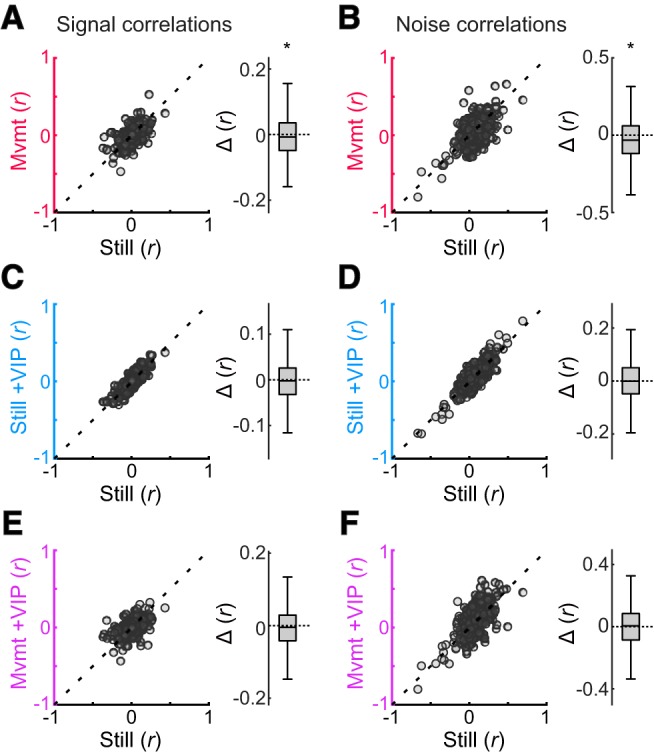
Coordinated network variability decreases in ACtx are during movement but is unaffected by VIP^+^ interneuron activation. ***A***, ***B***, Scatterplot summaries of signal and noise correlations for Still and Mvmt conditions (*n* = 441 neuron pairs). A difference box plot (Δ) appears to the right of each scatterplot representing Mvmt values subtracted from Still values. Box plot mid lines indicate medians, outer lines indicate 25th and 75th percentiles, and whiskers indicate the range of data points excluding outliers. Movement was associated with a small but significant reduction in signal correlations (mean decrease, 0.006), which reflect similarity in frequency tuning. A more substantial reduction was observed for noise correlations (mean decrease, 0.0245), which reflect coordinated activity resulting from factors other than the stimulus. ***C***–***F***, Scatterplot summaries of signal and noise correlations for Still and Still + VIP conditions (***C***, ***D***) and for Still and Mvmt + VIP conditions (***E***, ***F***). No significant differences from Still were observed during either VIP activation condition. *p < 0.05, signed-rank tests.

To our knowledge, changes in neuronal correlations have not been reported in connection with movement-related changes in firing patterns in ACtx. The inverse relationship between noise correlations and information content in VCtx during movement suggests that noise correlations might significantly increase during movement in ACtx, or alternatively, that signal correlations would more substantially decrease. We obtained support for neither of these straightforward hypotheses (Fig 5*A*,*B*). Instead, we found a significant reduction in signal correlations [*p* = 0.027, *r* = 0.074, WSR test; Still: mean = 0.007 (CI, −0.001 to +0.015), median = 0.007 (CI, 0.003–0.011); Mvmt: mean = 0.001 (CI, −0.008 to +0.011), median = 0.002 (CI, −0.003 to +0.009); mean change from Still = −0.006 (CI, −0.014 to +0.002)], but a more substantial reduction in noise correlations [*p* < 10^−4^, *r* = 0.146, WSR test; Still: mean = 0.051 (CI, 0.037–0.064), median = 0.045 (CI, 0.033–0.056); Mvmt: mean = 0.026 (CI, 0.010–0.041), median = 0.024 (CI, 0.001–0.037); mean change from Still = −0.024 (CI, −0.037 to −0.012)]. Qualitatively, these outcomes replicate trends reported by Dadarlat and Stryker (2017) in VCtx, although the effects were stronger in ACtx (signal correlations reduced by 0.003 in VCtx and 0.006 in ACtx; noise correlations reduced by 0.014 in VCtx and 0.024 in ACtx). Although additional study is warranted, this outcome implies that the effect of movement on coordinated network variability in ACtx and VCtx at least partially overlap. It also implies the existence of multiple, orthogonal consequences of locomotor activity that competitively shape information propagation and encoding efficiency in sensory cortex.

Consistent with our finding that VIP^+^ activation did not significantly influence information content, the difference between the Still and Still + VIP conditions (Fig. 5*C*,*D*) was nonsignificant for both signal [*p* = 0.302, *r* = 0.048, WSR test; Still: mean = 0.007 (CI, −0.001 to +0.015), median = 0.007 (CI, 0.003–0.012); Still + VIP: mean = 0.003 (CI, −0.005 to +0.011), median = 0.002 (CI, −0.004 to +0.006); mean change from Still = −0.003 (CI, −0.008 to +0.001)] or noise correlations [*p* = 0.573, *r* = 0.019, WSR test; Still: mean = 0.051 (CI, 0.037–0.063), median = 0.045 (CI, 0.033–0.057); Still + VIP: mean = 0.053 (CI, 0.039–0.068), median = 0.049 (CI, 0.040–0.059); mean change from Still = +0.002 (CI, −0.006 to +0.009)]. However, the difference was also nonsignificant between Still and Mvmt + VIP conditions (Fig 5*E*,*F*) for both signal [*p* = 0.174, *r* = 0.058, WSR test; Still: mean = 0.007 (CI, −0.001 to +0.015), median = 0.007 (CI, 0.003–0.012); Mvmt + VIP: mean = 0.002 (CI, −0.005 to +0.010), median = −0.000 (CI, −0.006 to +0.005); mean change from Still = −0.005 (CI, −0.012 to +0.004)] and noise [*p* = 0.343, *r* = 0.032, WSR test; Still: mean = 0.051 (CI, 0.038–0.063), median = 0.045 (CI, 0.033–0.056); Mvmt + VIP: mean = 0.057 (CI, 0.042–0.073), median = 0.052 (CI, 0.039–0.066); mean change from Still = +0.006 (CI, −0.007 to +0.019)] correlations. The latter outcome was unexpected in light of our earlier finding that the consequences of movement and VIP^+^ activation tended to sum linearly and highlights an additional minor exception to this general trend. It provides additional evidence that network variability and stimulus encoding dynamics (stimulus-evoked spike rates, information content, and efficiency) are not strictly interdependent.

## Discussion

Numerous studies have established that stimulus processing in sensory cortex is sensitive to a wide range of variables reflecting the state of the organism. This includes a large body of literature documenting substantial differences between anesthetized and awake preparations (Young and Brownell, 1976; Capsius and Leppelsack, 1996; Gaese and Ostwald, 2001; Szalda and Burkard, 2005; Ter-Mikaelian et al., 2007; Marguet and Harris, 2011; Haider et al., 2013). In awake subjects, considerable evidence has emerged that stimulus processing is strongly modulated by the behavioral context in which sounds occur (Polack et al., 2013; Eggermann et al., 2014; Erisken et al., 2014; Reimer et al., 2014; Schneider et al., 2014; McGinley et al., 2015a, b; Vinck et al., 2015). Our results are consistent with previous studies reporting a net decrease in stimulus-related firing by excitatory neurons in rodent ACtx during spontaneous movement (Schneider et al., 2014; Zhou et al., 2014). A similar attenuation of ACtx evoked responses has been observed in rodents, primates, and humans during related behavioral states including auditory task engagement (Otazu et al., 2009; Kuchibhotla and Bathellier, 2018), vocal production (Eliades and Wang, 2003, 2008; Flinker et al., 2010), and in response to self-generated sounds (Martikainen et al., 2005; Aliu et al., 2009; Mifsud et al., 2016; Rummell et al., 2016). Although response attenuation in self-generation paradigms is partially attributable to expectation-related feedback (Todorovic et al., 2011; Rummell et al., 2016), recent experiments suggest that it also depends at least in part on independent feedback generated by motor responses (Rummell et al., 2016). Thus, studies examining ACtx across a variety of behavioral contexts have routinely found that motor activity tends to reduce sound-evoked spiking. Expanding on these findings, we found that the reduction in stimulus-evoked spike rates during movement coincides with a loss of information about the stimuli (bits), which was further compounded by reduced encoding efficiency (bits per spike). These outcomes fit squarely with the recent finding that behavioral detection of auditory stimuli was significantly reduced during movement (McGinley et al., 2015).

These outcomes stand in contrast to results from numerous studies of movement-related changes in stimulus processing in VCtx, which have consistently observed increased evoked firing rates, information content, and encoding efficiency (Niell and Stryker, 2010; Dadarlat and Stryker 2017). Extending these findings, a recent study examined neural activity in higher-order visual cortical areas inferred from fluorescence change measures of cellular activity (Christensen and Pillow, 2017). Consistent with previous findings, stimulus information and response reliability increased during movement, although neural activity in these areas actually decreased. Congruent with enhanced processing at the cortical level, movement has also been shown to improve detection of low-contrast visual stimuli at the behavioral level (Bennett et al., 2013). As discussed previously (Dadarlat and Stryker, 2017), these observations are consistent with a shift in cortical state during movement to accommodate increased load on the visual system. Our findings extend this notion by suggesting that such a shift not only favors information processing within VCtx but also disfavors information processing in ACtx.

Several lines of evidence raise the possibility that such a difference may be better explained by a resource allocation shift in favor of spatial information processing during movement, rather than one strictly favoring the visual over the auditory pathway. First, recent experiments in rodent VCtx have found that increases in relative gain and information content during movement are not uniformly distributed across neurons with disparate receptive fields, but instead favor those tuned to high spatial frequencies (Mineault et al., 2016). VCtx is thus biased in support of heightened spatial acuity during movement, a finding hypothesized to correspond to the heightened spatial discrimination ability observed in human subjects during spatial attention tasks (Anton-Erxleben and Carrasco, 2013). Second, it has long been recognized that the firing rates of hippocampal place cells are positively correlated with running velocity, consistent with increased engagement of spatial circuitry during movement (McNaughton et al., 1983). Third, in cats performing an explicitly spatial auditory task, ACtx neurons exhibited significant increases in spatial sensitivity, despite a net decrease in evoked firing rates (Lee and Middlebrooks, 2011). Congruent with these findings, recent human neuroimaging data also indicate that auditory spatial information is elevated in ACtx during auditory spatial task engagement (van der Heijden et al., 2018). Finally, attenuated responses to nonspatial auditory stimuli have been observed in the hippocampus (i.e., outside the primary auditory pathway) during a sound self-generation paradigm (Rummell et al., 2016). Thus, the largely opposite consequences of motor activity on the auditory and visual pathways may reflect their differential contributions to spatial processing during movement. To further validate this hypothesis, additional studies are needed to examine the influence of movement on processing stimuli that vary along a spatial dimension within the auditory system and stimuli that vary along a nonspatial dimension within the visual system (Ungerleider and Mishkin, 1982; Kaas and Hackett, 1999).

As in previous studies of ACtx and other cortical areas, VIP^+^ interneuron activation tended to elevate stimulus-driven spike rates in our unit sample, which is consistent with the disinhibitory circuit model described in previous studies (Lee et al., 2013; Pfeffer et al., 2013; Pi et al., 2013; Zhang et al., 2014). Importantly, however, these spikes were apparently unrelated to differentiating the stimuli used in our study, resulting in zero net change in mutual information (bits). Thus, although they did not directly interfere with stimulus processing, they incurred costs in terms of encoding efficiency (Lennie, 2003). These outcomes are largely consistent with previous work examining changes in frequency tuning derived from pure tone responses (Pi et al., 2013). As in the present study, VIP^+^ activation elevated stimulus-evoked firing rates for the majority of cells. However, the tuning curves were augmented in an additive rather than multiplicative fashion, implying a shift without sharpened selectivity (see also Dadarlat and Stryker, 2017). The intersection of movement and VIP^+^ activation in our study was largely explained by their linear summation, as follows: VIP^+^ activation recovered the reduction in evoked spike rates during movement, but did not recover the loss in information content. Thus, evoked spike rates during movement were indistinguishable between the Still and Mvmt + VIP conditions, although information content and encoding efficiency were significantly reduced. These outcomes constitute an important departure from findings in VCtx, in which the effects of VIP interneuron activation studied so far largely reproduce the effects of movement, including stimulus-evoked firing (Fu et al., 2014) and instantiation of adult cortical plasticity (Fu et al., 2015).

Because enhanced processing in VCtx during movement occurs in tandem with substantially reduced noise correlations (Dadarlat and Stryker 2017), we hypothesized that the diminished information content and worse encoding efficiency in ACtx might be associated with an increase in noise correlations, or alternatively, a more substantial decrease in signal correlations. Contrary to these expectations, we observed a small decrease in signal correlations but a larger decrease in noise correlations, in parallel to VCtx. These outcomes suggest that the influence of movement on cortical stimulus processing and coordinated network variability may be largely independent from one another. In any case, whereas the benefits of reduced noise correlations add to increased gain in VCtx, in ACtx they are overwhelmed by the suppression of stimulus-related firing.

Potential differences between naturalistic and experimental manipulation of interneuron activity should be considered when interpreting the results of the current and previous studies in which optogenetic or pharmacological techniques were used to artificially influence targeted subpopulations of VIP^+^ interneurons. Importantly, however, the basic effects of naturalistic VIP^+^ interneuron recruitment have been faithfully reproduced by optogenetic activation in several experiments examining stimulus encoding and plasticity in VCtx (Fu et al., 2014, 2015). Thus, although there is ultimately no “ground truth” for establishing the strict equivalence of optogenetic and naturalistic effects, these parallel outcomes suggest they will likely lead to the same fundamental influences on cortical processing in the context of the present set of experiments and related work in VCtx. Nevertheless, although qualitatively similar, we caution against drawing conclusions about potential quantitative differences between optogenetic and naturalistic conditions from the current experiments and previous VCtx publications. Such differences would undoubtedly depend on complex interactions among parameters such as light intensity and movement velocity, which have not been examined in the current or previous studies.

**Figure 6.**
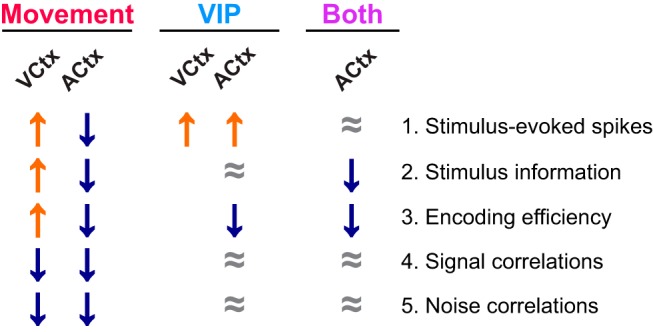
Summary of the influences of movement and VIP^+^ interneuron activation in visual and auditory cortices. Relative to Still: ↑, increase; ↓, decrease; ≈, no change; blank entries, presently unknown. Note that these changes are intended to represent outcomes on balance, despite heterogeneity among individual neurons.

In summary, movement and VIP interneuron activation have dissociable consequences for stimulus-evoked spike rates, stimulus information, and encoding efficiency in mouse ACtx. With only a few exceptions, the consequences of movement in ACtx appear to be largely opposite of those in VCtx (summarized in Fig. 6), a finding that may reflect their differential contributions to spatial perception likely to be differentially recruited during movement. A more complete understanding of the conditions governing these differential influences on sensory processing, as well as the circuitry underlying these changes, could have important implications for the perspectives of both basic science and clinical practice. In the visual system, several experiments have demonstrated that pairing visual stimulation with movement is sufficient to instantiate several forms of lasting adult cortical plasticity (Kaneko et al., 2017). This includes recovery from amblyopia induced during the critical period (Kaneko and Stryker, 2014), an outcome replicated by optogenetic activation of VIP^+^ interneurons (Fu et al., 2015). Although research extending these findings to human applications has just begun, several initial studies have found that physical activity is indeed capable of inducing at least short-term plasticity in adult visual cortex (Lunghi and Sale, 2015; Perini et al., 2016; Bullock et al., 2017). Such approaches contribute to an emerging class of noninvasive brain plasticity-based therapeutics currently being developed in the service of recovery from brain injury; for treating cognitive, neurologic, and psychiatric dysfunction; and for preventing pathologic aging of the brain (Merzenich et al., 2014). The present results suggest that any plasticity induced in ACtx by pairing movement with the sound types studied so far, if any, is likely to differ from that observed in VCtx. If so, the discovery of possible alternative paradigms better suited to harnessing adaptive movement-related plasticity in ACtx will require future study.

## Synthesis

Reviewing Editor: Tatiana Pasternak, University of Rochester Decisions are customarily a result of the Reviewing Editor and the peer reviewers coming together and discussing their recommendations until a consensus is reached. When revisions are invited, a fact-based synthesis statement explaining their decision and outlining what is needed to prepare a revision will be listed below. Note: If this manuscript was transferred from JNeurosci and a decision was made to accept the manuscript without peer review, a brief statement to this effect will instead be what is listed below.

The reviewers agreed that the revised manuscript addressed many of the concerns raised in their reviews. However, they raised a few other issues that still must be addressed before the paper can be published in eNeuro. These are summarized below.

1. Please include in the manuscript the discussion of the interpretation of the VIP neurons optostimulation that was included in your rebuttal letter.

2. Please compare size effects and spike rate changes in VCtx to the present work in ACtx.

3. Please provide the information (mean, median and 95% confidence interval) in the results or figure legends about specific spike rates.

4. Please change SEM to 95% confidence interval for all the results

5. Please address the comment by Reviewer 2 about your citation of Christensen and Pillow paper.

----------------

REVIEWER 1.

ADVANCES IN THE FIELD:

The authors provided new findings that movement decreased the mutual information between auditory stimuli and cortical activity, as well as encoding efficiency. These findings are in contrast with previous reports of the effects of movement on visual cortical information processing, and post interesting questions for future studies.

COMMENTS TO THE AUTHORS

The authors have revised their manuscript to address the concerns raised in the previous round of review. Although the scope of this study remains limited, the current findings are interesting and suggest important questions for future research.

One remaining issue is that the authors discussed the caveats related to the interpretation of VIP neuron opto-stimulation results in the rebuttal, but did not incorporate them into the manuscript. These discussions, together with appropriate comparison to previous studies cited in the rebuttal, are important for the general readers to evaluate the difference between movement and VIP neuron stimulation induced effects and should be included in the manuscript.

REVIEWER 2.

ADVANCES IN THE FIELD:

Small statistically significant changes in firing rate and MI (selectivity) with movement and VIP activation. Useful comparison to prev V1 data.

STATISTICS

Almost correct, but the CIs I requested should be added. (Effect sizes in this paper are small, so statistical analyses are especially critical to get correct)

COMMENTS TO THE AUTHORS

Comments in order of rebuttal letter:

- Please add at least a few sentences comparing effect sizes (i.e. changes in spike rate, changes in MI in bits) in VCtx to the present work in ACtx. Are the spike rate changes much larger or smaller in one data set or the other? If so, why?

- What are the firing rates before and after change for Fig. 2C, mean, median, and 95% CI? (“a significant decrease in stimulus-evoked spikes (Fig. 2C) was observed for BS units (mean decrease 64+-23%, p = 0.023, r = 0.191, WSR test).”) Same, for Fig. 3C? This is important because these are difficult to discern from current figure panels. Please add these numbers to the Results text (or figure legend). Please include same metrics for information (bits) comparisons throughout as well.

- Percent changes were added throughout Results. The percent change means are fine, but please change SEM to confidence intervals, since the main statistical tests reported now are not done on the percentages (suggest 95% CI via bootstrap).

- Thank you for revising for clarity wrt “encoding”, now improved.

- Re: Christensen and Pillow BioRxiv, if you choose to keep citation, please note in text that in many cases (at least in the version of the manuscript now on the site), the work uses dF/F changes, and does not measure or calculate firing rates. Several factors could create changes in calcium or fluorescence (and impact reliability, and thus information) that do not appear in spike recordings, such as neuropil responses or movement-related ACh inputs.
